# Preliminary study on the effects of treatment for breast cancer: immunological markers as they relate to quality of life and neuropsychological performance

**DOI:** 10.1186/s12905-020-00971-1

**Published:** 2020-05-20

**Authors:** Michael J. Boivin, Geoffrey P. Aaron, Nathan G. Felt, Lance Shamoun

**Affiliations:** 1 Department of Neurology & Ophthalmology, Michigan State University, East Lansing, USA; 2 Department of Psychiatry, 909 Wilson Road, Rm 327, West Fee Hall, East Lansing Michigan, East Lansing, MI 48824 USA; 3 Children’s Healthcare of Atlanta, Atlanta, GA USA; 4 Digital Experience Design (DXD), San Francisco, CA USA; 5 Department of Human Medicine, Michigan State University, East Lansing, MI USA

**Keywords:** Breast cancer, Quality of life, Immunology, Neuropsychology, Emotional wellbeing, Spirituality

## Abstract

**Background:**

Immunological biomarkers were related to quality of life and neuropsychological performance in women recently diagnosed with breast cancer through the first six months of treatment. A comparison group of breast cancer survivors in remission were also evaluated.

**Method:**

Twenty women newly diagnosed with breast cancer and 26 breast cancer survivors at least a year after treatment were evaluated four times over a course of six to 8 months. The assessments included quality-of-life, emotional and spiritual well-being, sleep quality, computerized neuropsychological performance, and cytokine immunology biomarkers using flow cytometry. The principal immunological markers examined were the CD4+, CD8+, and CD16+ counts.

**Results:**

Although equivalent at enrollment, active treatment women reported higher anxiety, depression, poorer quality-of-life, and poorer processing speed and accuracy on memory, logical processes, and coding neuropsychological tasks. They also had significantly higher CD8+ and CD16+ cell count levels during treatment over the next six to eight months than comparison group women in remission. Women undergoing chemotherapy as well during treatment phase also had a significant decline in CD4+ counts. Higher percent CD8+ levels during treatment was associated with poorer quality of life and more depression, while higher CD4+ and CD8+ were associated with poorer neuropsychological memory and processing speed performance.

**Conclusion:**

Significant increases in CD8+ is a sensitive biomarker of a broad range of poorer quality-of-life and neurocognitive functioning outcomes during breast cancer treatment, especially in women undergoing chemotherapy. Quality of life should be monitored in breast cancer patients and psychosocial support made available as a standard of care.

## Background

The treatment of breast cancer is a complex process that involves more than treating a tumor. The women undergoing treatment deal with many levels of psychological events, including fighting a life-threatening disease with toxic therapies, changes in physical appearance, and managing the intricacies of the complex medical system. All the while attempting to continue the normalcy of life [1].

For women, stress and mood disturbance arising from the breast cancer journey can significantly modify immunological response during the course of treatment [2]. For example, the psychospiritual profile of women can impact on the efficacy of cancer chemotherapy and its immunological impact [3]. Maes et al. (1992) found that in major depression that there was an increase in CD4/CD8 T-lymphocytes, [4] and Sephton et al. (2009) found that women displaying more depressive symptoms had weaker immune system response [5].

Spiritual well-being and coping as well predict CD4-cell preservation in immune-compromised patients [6]. Subsequently, there have been a number of spiritually-based interventions used to evaluate their psychoneuroimmunology benefit in breast cancer patients [7]. Bauer-Wu and Farron (2005) observed in their research that in breast cancer survivors that a positive correlation exists between a survivor’s meaning in life and spirituality [8]. Likewise, a higher level of spirituality and self-forgiveness has been found to be predictors of a better quality of life and less mood disturbances in breast cancer patients. This is because improvement in quality of life, as with their meaning in life, could lead to the increase of immune functions [9].

The goal of this study was to evaluate the relationship between emotional well-being (EWB), social support, quality of life (QoL), and spiritual well-being (SWB) -- with several key immunological biomarkers (CD4, CD8, CD16 positive cells). Since these interrelationships can impact on neurocognitive functioning, [10] we will also evaluate the relationship between immunological biomarkers and neuropsychological performance on a computerized screening battery. Thus, in this preliminary study we will assess the relationship between biomarkers of the immune system, and questionnaire measures of emotional well-being (EWB), social support, quality of life (QoL), spiritual well-being (SWB), and neuropsychological functioning. We will evaluate these relationships longitudinally, beginning at diagnosis for early-stage breast cancer patients, and continuing through the course of treatment. These interrelationships will be compared to those of a group of breast-cancer survivors who have completed treatment at least a year earlier and are in remission.

In their review of the role of the biomedical in exploring the role of spirituality in breast cancer research, Boivin and Webb (2011) describe several levels whereby psychoneuroimmunology can interface with spirituality in a women’s medical journal through breast cancer [11]. The best-known pathway between the immune and nervous systems involves neural signals passing from the periphery to the brain through the vagus nerve. These innervate immunological pathways in the gut, spleen, thymus, and lymph nodes, [12] sending afferent messages from the innervated organs to the brain. Spirituality can be a powerful modifier of how psychosocial stress impacts upon this brain/immunological two-way interface [12]. To illustrate, Tai Chi Training and Spiritual Growth Groups can enhance immunological resilience in the face of breast cancer disease and its treatment with chemotherapy [13, 14]. We hypothesize in the present study that positive psychosocial functioning, quality of life (including spiritual well-being), and immunological biomarkers indicative of a more robust response (CD4, CD8, CD16 positive cells) will be positively related to one another.

We chose CD4 cells, also called T4 cells, because they are immunological “helper” cells that effectively respond to treatment for depression in women. CD8 cells, (T8 cells), are “suppressor” cells and complete the immune response to chronic stress and its response to psychosocial support. CD8+ cells can also be “killer” cells that kill cancer cells and other cells that are infected by a virus. CD16 cells are responsible for antibody-dependent cellular cytotoxicity and respond to emotional wellbeing and chronic stress in patients [15–18].

Therefore, in the present study our principal outcomes of interest pertain to the domains of psychosocial functioning (including social support), emotional wellbeing, quality of life (including spiritual well-being), T cell immunological biomarkers (CD4, CD8, CD16), and breast cancer disease symptom severity. We also use a computerized neuropsychological screening assessment to evaluate neurocognitive performance in important domains (e.g., attention, working memory, learning and recall, visual-spatial analyses, planning/reasoning, problem solving, distractibility and executive functions).

 These will be assessed at the point of diagnosis, at multiple points during treatment, and 2 months following completion of radiation/chemotherapy treatment. Outcomes for the active breast cancer treatment group were compared to breast cancer survivors (at least a year out of treatment) enlisted from a breast-cancer survivor support group that met every couple of weeks at the same hospital as the active treatment women. It is important to evaluate the relationship between immunological biomarkers and overall quality of life (emotional wellbeing - EWB, spiritual wellbeing - SWB, psychosocial support) as impacted over the course of breast cancer diagnosis and treatment because of how quality of life can modify the impact of treatment on immunological response (see Boivin and Webb, 2011 for a more detailed model of these causal interactions) [11]. Likewise, we use a computerized neuropsychological screening battery to evaluate the effects of breast cancer diagnosis and treatment on these neurocognitive processes, because of how they might partially mediate overall quality of life [11].

## Methods

### Participants

IRB approval for this study was obtained from both Indiana Wesleyan University and Lutheran Hospital (Fort Wayne, IN) and informed written consent was obtained from all women prior to study participation. All patients were recently diagnosed with Stage 1 to 3a breast cancer (using the Tumor, Node, Metastasis (TNM) Stage Grouping System) at the Lutheran Hospital Breast Center and Women’s Health clinic in Fort Wayne, Indiana (see Table 1). They were contacted over a two-month period, and 20 of them (66%) agreed to participate. Following initial surgery (lumpectomy and/or mastectomy) all participating women (active treatment group) in the present study underwent radiation therapy (*N* = 20). Depending on the staging and recommended treatment guidelines, most active treatment women then had chemotherapy (Cyclophosphamide, Methotrexate, Fluorouracil or CMF) or Taxotere (Docetaxel) (Table 1). These were all women in the active treatment group with a positive lymph node biopsy during surgery (*N* = 9) *as per* the hospital oncology department standard of care for breast cancer at that time. Unfortunately, the molecular subtype of breast cancer (e.g., triple negative, HER2+, HR+) was not available for the active patient or breast cancer survivor (remission) groups in the present study. Though data on molecular subtype of breast cancer is not available, some patients in both groups received tamoxifen (Table 1), suggesting a hormone-dependent (luminal) disease. Those not receiving the drug most likely belonged to HER2-positive or triple-negative subtype.

**Table 1:** Demographic description of present study samples

Characteristic	Remission group ***N*** = 26	Active treatment group ***N*** = 20	***P***-value for between-group comparison
	**Mean (St Dev)**	**Mean (St Dev)**	
**Age**	55.7 (10.21)	55.2 (12.26)	0.87
	**N (%)**	**N (%)**	
**Education**			0.38
High school or less	6 (23%)	8 (40%)	
Some college	8 (31%)	4 (20%)	
College degree	5 (19%)	6 (30%)	
Post-graduate work or degree	7 (27%)	2 (10%)	
**Marital Status at Enrollment**			0.42
Single, never married	2 (8%)	1 (5%)	
Married	17 (65%)	16 (80%)	
Separated	0 (0%)	1 (5%)	
Divorced	5 (19%)	2 (10%)	
Widowed	2 (8%)	0 (0%)	
**Employment Status at Enrollment**			0.45
Working full-time	13 (50%)	6 (30%)	
Working part-time or at home	6 (24%)	4 (20%)	
Retired	5 (19%)	7 (35%)	
Student	0 (0%)	1 (5%)	
Disabled	1 (4%)	2 (10%)	
**Breast Cancer Stage at Enrollment: Tumor, Node, Metastasis (TNM) Stage Grouping System**			NA
Stage 0 Noninvasive ductal carcinoma in situ (DCIS)		1 (4%)	
Stage 1		5 (25%)	
Stage 2 (a & b)		11 (55%)	
Stage 3 (a)		3 (15%)	
Stage 4		0 (0%)	
**Type of chemotherapy by completion of study**			0.047
Cyclophosphamide Methotrexate Fluorouracil (CMF)	13 (50%)	3 (15%)	
Taxotere (Docetaxel)	5 (19%)	6 (30%)	
None	8 (31%)	11 (55%)	
**History of surgery at time of enrollment**			0.034
Lumpectomy	10 (38%)	14 (70%)	
Mastectomy	16 (62%)	6 (30%)	
**History of radiation treatment by completion of study**			0.0012
Yes	10 (38%)	20 (100%)	
No	16 (62%)	0 (0%)	
**History of tamoxifen by completion of study**			0.047
Yes	18 (69%	8 (40%)	
No	8 (31%)	12 (60%)	

*P value for age is from a Student’s *t* test. *P* value for all remaining descriptive measures is from a Chi Square test or categorical frequencies by group (Breast cancer remission versus active treatment). NA: not applicable

Women were excluded if their medical chart review revealed significant neuropsychological risk from a history of central nervous system disease or infection (e.g., meningitis, HIV, stroke), seizures, prior cancer diagnoses, or traumatic brain injury or accident.

Through a breast cancer survivors support group meeting monthly at the Lutheran Hospital Breast Center, a comparison group was enrolled. These were all women who had completed treatment for breast cancer at least a year previously (*N* = 26), including chemotherapy (*N* = 18). All present women in the active treatment group given chemotherapy completed the full course of treatment in accordance with the American Cancer Association guidelines for early stage breast cancer, either CMF or Taxotere. The cancer survivor comparison group members were also excluded if a medical history revealed any of the exclusion neuropsychological risk factors as noted above for the active treatment group. Although the breast cancer survivor comparison group could report their treatment history, we did not have access to their original disease stage at diagnosis. The two groups were equivalent on age and education, all were Caucasian and the majority were married, employed, and with at least some college education (Table 1).

#### Questionnaires

Zung Self-Rating Depression Scale [19, 20]. The Zung Self-Rating Depression Scale (Zung SDS) is a self-administered 20-item questionnaire that includes a variety of statements associated with depressed moods and is a helpful tool used to assess depression in individuals. The inventory looks at various symptoms of depression such as insomnia, fatigue, suicidal thoughts, and anxiety. The 20 items are based on a Likert scale and the four possible responses range from “None or little of the time” to “Most or all of the time.” The higher the score, the more depressed the respondent.

The State-Trait Anxiety Inventory [21–23]. The State-Trait Anxiety Inventory (STAI) is a measure that looks specifically at anxiety. It measures a person’s current anxiety (state) and characteristic anxiety (trait). The full STAI is a 40-item self-report anxiety inventory, but a shortened version of the STAI consisting of five items was used in this study [21, 22]. The higher the score, the more anxious the respondent.

Hope Quality of Life Scale – Breast Cancer Patient Version [24–27]. This version of the Quality of Life Scale (Ferrell, Dow, & Grant, 1995) is based on previous versions created by the City of Hope National Medical Center. The assessment is a 46-item questionnaire that looks at the four domains of quality of life: physical well-being, psychological well-being, social well-being, and spiritual well-being. This instrument has been validated and reliability has been insured over multiple uses in hospital studies [26–28]. The higher the score, the better the quality of life.

Pittsburgh Sleep Quality Index [29, 30]. A modified version of the Pittsburgh Sleep Quality Index (PSQI) was used to evaluate sleep quality in the patients. The PSQI is broken down into seven parts for scoring. They are subjective sleep quality, sleep latency, sleep duration, habitual sleep efficiency, sleep disturbances, use of medication, and daytime dysfunction. The higher the score on these seven components the more likely the person is to have sleep disturbances. We also included the Sleep Hygiene Likert Scale optional items with this questionnaire.

Functional Assessment of Cancer Therapy - General (FACT-G) [31]. This assessment is a comprehensive compilation of questions which measures health-related quality of life (QOL) in patients with cancer and other chronic illnesses. This study used the FACT-G in conjunction with the subscale for assessing specific problems related to anemia in cancer patients (FACT-An), a subscale which is represented by questions addressing the cardinal symptom of anemia, which is fatigue. This measure also helped gauge the impact of cancer treatment, such as chemotherapy, on other measures of emotional, psychological, and spiritual well-being. For two of the scales (Physical, Functional) higher scores indicate poorer functionality. For the other two scales (Social/Family Well-being, Emotional Well-being), higher scores indicate higher well-being.

Fatigue Symptom Inventory (FSI) and the Multidimensional Fatigue Symptom Inventory (MFSI) [32–34]. The MFSI (Hann, et al., 1998) is a 14-item self-report measure designed to measure the daily patterns of fatigue including intensity, frequency, and impact on overall quality of life. The MFSI (Stein, Martin, Hann, & Jacobsen, 1998) is an 83-item self-report measure that evaluates the principal manifestations of fatigue; and includes the subscales of global, somatic, affective, cognitive, and behavioral manifestations of fatigue. These instruments have been validated in a variety of clinical contexts with cancer patients, and provide for a sensitive overview of the impact of the disease on patients’ overall quality of life as well as activities of daily living (ADL) [32, 34]. Lower scores indicate less intense symptoms of fatigue, so the lower the score the better.

Spiritual Beliefs Inventories (SBI) [35, 36]. In a landmark publication which gained credibility for the inclusion of spiritual well-being assessment within the broader evaluation of quality of life (QOL) issues in psycho-oncology research, Holland and her co-workers (Holland et al., 1998) developed and published the Spiritual Beliefs Inventory. Unlike previous spiritual well-being inventories (e.g., the Spiritual Well-Being Scale in Paloutzian and Ellison, 1991) used in health-related research (e.g., Carson & Green, 1992; Kaczorowski, 1989), the SBI does not rely exclusively on spiritual and religious beliefs, but also includes a social support measure derived from involvement with church or religious groups. The result of their efforts is a well-validated 15-item questionnaire that is a brief, yet robust, measure of the more universal aspects of religious, spiritual, and religious community social support coping with a life-threatening illness as well as the subsequent QOL impact. Also included was the Paloutzian and Ellison 20-item Spiritual Well-Being scale (SWB) that was developed to look at just spiritual and religious beliefs [37]. For both instruments, a higher score indicates a stronger sense of spiritual wellbeing.

Bottomley Social Support Breast Cancer Scale [38–40]. This is a cancer-specific questionnaire that is well validated in the clinical cancer-care context and also quick and easy to use in a busy clinical environment. The Bottomley Social Support Scale has good construct validity with the Hospital Anxiety and Depression Scale. It is reliable, allowing medical and support staff to assess the levels of social support and implement any appropriate social support interventions and sensitive to the duress of breast cancer treatment and care [41]. The higher the score, the less the perceived social support on the part of the respondent.

Automated Neuropsychological Assessment Metric (ANAM) [42]. This is a computerized neuropsychological assessment battery developed by researchers at the Walter Reed Medical Center for use in human performance factor studies (e.g., neuropsychological effects of fatigue, chronic stress, sleeplessness, toxic exposure). It takes about 45 to 60 min to complete for the average adult. The components of the assessment are derived from well-validated brain/behavior tests such as those encompassed in the Halstead-Reitan Neuropsychological Assessment Battery and the Wechsler Adult scales for both intelligence and memory. The principal measures that we were taken from this assessment pertain to executive functioning and problem solving (Tower of Hanoi Task, Symbolic Logical Reasoning Test), encoding and memory, and a continuous performance task measure of attentional capacity and response time. Furthermore, the ANAM battery has been validated in the hospital or clinic setting [42–44]. For all of the ANAM tests, the higher the score the better the performance with the exception of simple reaction time.

#### Flow Cytometry measures derived from blood cells and plasma assay

Twenty-five ml blood samples were drawn (2 × 10 ml Na-heparin, 1 × 5 ml clot) immediately before the computerized questionnaire and ANAM assessment at each of our four study assessment points. Blood was placed in heparinized tubes and was evaluated for hematocrit (percent of red blood cells) and total plasma protein (g/dl). Standard Wright’s staining procedure was used to obtain complete blood counts (CBC). The heparinized samples were then separated into the leukocyte and erythrocyte fractions by Histopaque-1077 gradient centrifugation. FITC-conjugated murine monoclonal antibodies were used to label and measure total T cell count (CD3), helper T cell (CD4+), cytotoxic T cell (CD8+), B cell (CD19+), and NK cell (CD16+) using flow cytometry. Each of these assays was conducted at the Indiana Wesleyan University (IWU) research laboratory facility under the direction of Burton Webb. To accomplish this, blood specimens at room temperature were transported from the Lutheran Hospital Fort Wayne study site (about a 1-h drive) within 24 h of the blood draw by an IWU student lab assistant trained in the safe handling of biohazard material, employed by Dr. Webb in his lab on a work-study program. Upon receipt at IWU, the blood specimens were immediately processed and placed in deep freeze frozen storage (− 70 °C) for later analysis. Dr. Webb, who at the time taught immunology to first-year medical students at Indiana University-Purdue University Indianapolis (IUPUI) Medical School-Muncie, supervised all immunology specimen processing and assay procedures directly in his laboratory at Indiana Wesleyan University, Marion.

#### Study procedure and rationale for immunological response measures derived from blood cells and plasma assay

Neuman and colleagues documented a relationship between immunological response and mental health [45]. A protocol similar to theirs for assessing the integrity of immunological response as it relates to psychological, emotional, neuropsychological, and spiritual well-being in the breast cancer patients was used. Blood samples were drawn immediately before the ANAM (computerized neuropsychological assessment) battery. Blood placed in heparinized tubes were evaluated for microhematocrit % (percent of red blood cells) using a centrifuge; and plasma protein values (g/dl) using a standard refractometer were determined from these samples. A radioimmunoassay kit was used to measure plasma cortisol (a stress hormone emitted by the adrenal cortex) and Toxicity-preventing activity (TxPA) (a serum albumin factor correlated with cardiovascular risk), along with lipid density measures. Standard staining procedures were used to obtain total white blood cells (WBC) as well as specific types of WBC important in combating disease and infections (mononuclear cells or lymphocytes, neutrophils, and monocytes). Fluorescein-labeled murine monoclonal antibodies were used to label and measure CD3, CD4, CD8, CD19, and CD16/CD56 lymphocytes using fluorescence-activated cell sorting (FACS) scan. NK (natural killer) white blood cells were also measured, since these provide protection against cancer cells and are related to blood vessel constriction following a stressor condition (Neumann & Chi, 1999). These assays were completed at the Indiana University Medical School Muncie Center for Medical Education immunology laboratory.

### Procedure

**Fig. 1:**
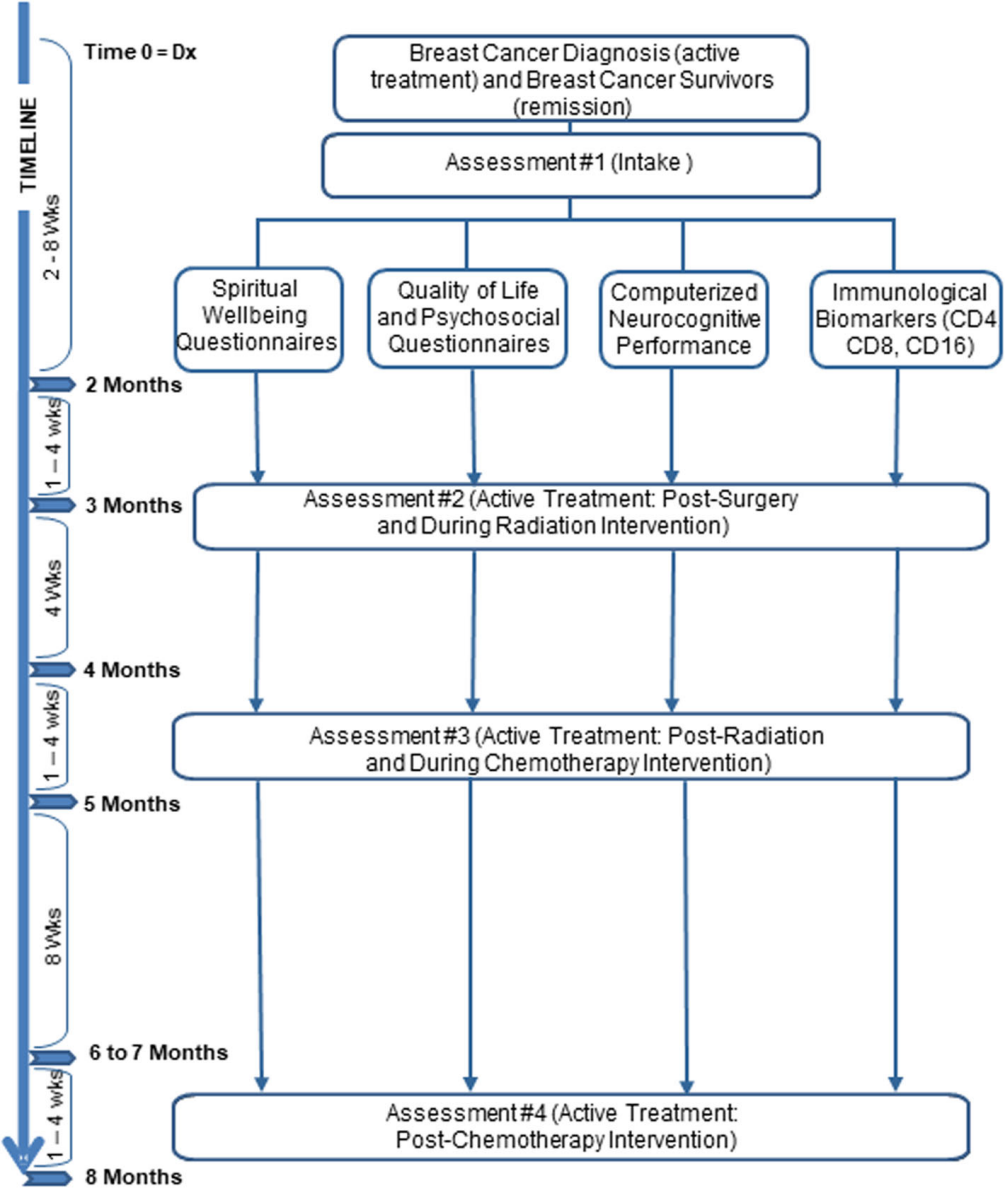
legend. The approximate timing of the 4 principal assessment domains (Spiritual Well-being, Quality of Life, Computerized Neurocognitive Performance, Immunological Biomarkers) is depicted for both the active treatment and remission (breast cancer survivor) groups in the present study. Timing of the assessments coincided with the approximate course of treatment from diagnosis/enrollment (Assessment 1), initial surgery and radiation treatment (Assessment 2), continuation/completion of radiation and initiation of chemotherapy (if needed) (Assessment 3), and completion of radiation and/or chemotherapy if needed (Assessment 4)

For the newly diagnosed breast cancer group, the first assessment point was at enrollment, within the first few weeks of diagnosis, usually following lumpectomy surgery and just before radiation treatment. The second assessment point was generally after surgery and during or just after radiation therapy, usually about one to 2 months after initial diagnosis for the active treatment group and enrollment for the remission breast cancer survivor group (Fig. 1). The third assessment point was at 2 months after treatment had started (after the conclusion of radiation therapy and in the initial stages of chemotherapy if recommended). The fourth assessment point occurred a month following completion of all chemotherapy if recommended. This was usually at the six- to eight-month point for the active treatment group and about 6 months following enrollment for the remission group. The second, third, and fourth assessments occurred 2–3 days after treatment (radiation and/or chemotherapy).

For the breast cancer survivor comparison group, the assessments occurred at enrollment, 1 month, 2 months, and at 6 months. The completed surveys and computerized ANAM neuropsychological assessment were completed in a quiet counseling room at the Women’s Cancer Center at Lutheran Hospital. Each blood drawn was completed by a registered nurse immediately following the assessments. Specimens then were processed at Muncie Center for Biomedical Education, where immunological biomarkers were assayed using flow cytometry.

### Analysis plan

Descriptive statistics at enrollment for active treatment breast cancer group and the breast cancer post-treatment (remission) comparison group not on active treatment, were compared using item averages for all questions (reverse coded where necessary) for each of the questionnaire measures (psychosocial, emotional wellbeing, quality of life, spiritual wellbeing, physical symptoms of cancer treatment). Statistically significant differences between the two groups were compared for these measures at *p* < 0.05 using an independent samples Tukey *t* test (nonparametric). The ANAM neuropsychological performance measures were computed automatically as age- and gender-based standardized scores using the ANAM norms built into the software package for American adults. Adjusting for age and years of formal education at enrollment (diagnosis), partial correlation coefficients were computed for the emotional wellbeing, quality of life, physical and health symptoms, and spiritual wellbeing measures when correlated with the immunology T cell biomarkers (CD4, CD8, CD16) for the active treatment and for the cancer survivor comparison groups separately. The most significant correlations were visually depicted in a scatterplot in the study figures.

Active treatment women were also compared across principal outcomes based on type of treatment prescribed (chemotherapy and radiation versus radiation only). For the active treatment subgroup comparisons (chemotherapy and radiation versus radiation only), an ANOVA repeated-measures analysis was used to evaluate whether a significant group by time interaction effect was apparent, indicating a greater degree of change over time for one treatment subgroup compared to the other. The most significant questionnaire-based (psychosocial support, quality of life, emotional wellbeing, spirituality, physical symptoms) time by treatment subgroup interaction effects was then depicted in the form of visual plot. The same was done with the most significant time by treatment subgroup interaction effect for the immunology biomarkers (CD4, CD8, CD16). IBM SPSS statistical software version 21 was used for all analyses, graphs, and plots [46].

## Results

**Fig. 2:**
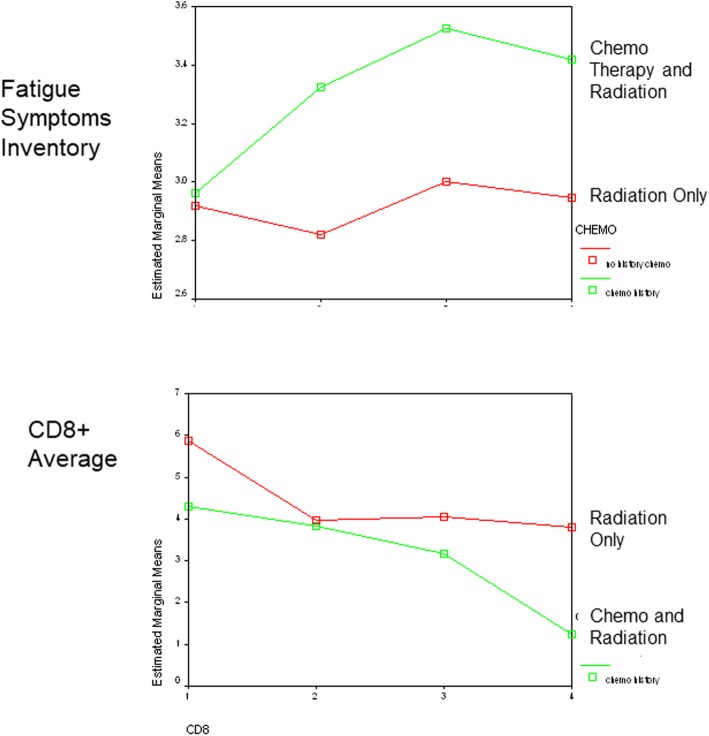
legend. Average mean item scores (adjusted for age at enrollment) are presented for the active treatment group of women, comparing those on chemotherapy and radiation (green line plot) versus those on radiation only (red line plot). Adjusted item averages by group are presented at 1) diagnosis/enrollment, 2) completion of radiation (~ two months post diagnosis), 3) completion of chemotherapy (~ 4 to 6 months post diagnosis), and post treatment (~ 6 to 8 months following diagnosis). The upper graph is for the most significant repeated measure statistical group by time interaction effect (Fatigue Symptoms Inventory) among the principal questionnaire outcome domains. The lower graph is for the most significant group by immunology biomarker outcome group by time interaction effect (CD8)

Active breast cancer and survivor comparison groups were comparable on age (Mean = 55.2 versus Mean = 54.7 years), post high-school formal education (Mean = 5.4 versus Mean = 4.5 years) (Table 1), and on all quality-of-life baseline questionnaire measures (Tables 2 and 3). They were also comparable on all the ANAM neuropsychological performance measures (listed in Table 4 but not presented). Active treatment women undergoing both radiation and chemotherapy (*N* = 9) had greater number of Fatigue Symptoms after the initiation of treatment compared to active treatment women undergoing radiation only (*N* = 11) (Fig. 2 upper). Likewise, CD8 levels decline throughout the treatment period for active treatment women undergoing both radiation and chemotherapy, compared to radiation treatment only (Fig. 2 lower).

**Table 2:** Descriptive statistics at enrollment for active treatment breast cancer group and the breast cancer post-treatment (remission) comparison group. Item averages are presented for all questionnaire measures. There are no statistically significant differences between the two groups on any of these measures at *p* < 0.05 for independent samples Tukey *t* test

	Post-Treatment Comparison Group- Remission			Breast Cancer Active Treatment Group - Diagnosis		
	N	Mean	Std. Deviation	N	Mean	Std. Deviation
Zung Depression Scale	26	1.71	.41	20	1.77	.44
State-Trait Anxiety Inventory	26	1.66	.53	20	1.74	.63
Hope Quality of Life (QoL) Breast Cancer Scale (all items)	26	5.24	.71	20	5.24	1.15
Hope QoL Physical Well-being	26	2.87	1.58	20	2.65	1.92
Hope QoL Psychological Well-being	26	7.17	1.00	20	6.71	1.48
Hope QoL Treatment Well-being	26	5.56	1.20	20	5.42	1.65
Hope QoL Fear and Anxiety Well-being	26	4.33	2.45	20	4.75	2.68
Hope QoL Treatment Well-being	26	3.34	1.47	20	4.06	1.68
Hope QoL Spiritual Well-being	26	7.82	1.66	20	7.73	1.66
Paloutzian and Ellison Spiritual Well-being	26	5.28	.78	20	5.20	.65
Holland Spiritual Beliefs Inventory (all items)	26	2.49	.57	20	2.42	.53
Fatigue Symptom Inventory	26	3.03	1.75	20	2.89	2.17
Pittsburgh Sleep Quality Index	25	1.65	.81	20	1.99	1.17
Sleep Hygiene Likert Scale	26	4.57	.47	20	4.34	.71
Functional Assessment of Cancer Therapy (FACT) – General (item average for all items)	26	1.49	.25	20	1.58	.44
FACT Physical Well-being Scale	26	.67	.44	20	.84	.88
FACT Social/Family Well-being Scale	26	3.26	.65	20	3.33	.62
FACT Emotional Well-being Scale	26	.62	.51	20	.79	.76
FACT Functionality Well-being Scale	26	3.03	.67	20	2.86	.81
FACT Social Concerns Well-being Scale	26	.93	.53	20	1.04	.79
Bottomley Cancer Social Support (item average: all items)	25	1.76	.69	20	1.60	.50

**Table 3:** Partial correlation coefficients for questionnaire measures for active treatment patients

	**Partial Correlation Coefficients – Controlling for Age & Education**					
	Two Months into Treatment			After Treatment Completion		
	CD4	CD8	CD16	CD4	CD8	CD16
**Hope Quality of Life (QoL) Breast Cancer Scale**						
Total Quality of Life (all)	−.40	−.62*	−.33	−.56*	−.27	−.46
Physical Well-being	−.16	−.70*	−.55	−.47	−.47	−.40
Psychological Well -being	.28	.65*	.22	.12	−.10	−.21
Treatment Well-being	−.43	−.44	−.46	−.24	.13	−.13
Fear and Anxiety Well-being	−.35	−.74**	−.12	−.49	.03	−.21
Social Concerns	−.38	−.74**	−.25	−.44	−.22	−.34
Spiritual Well-being	.29	.52	.19	.13	−.22	−.01
**Functional Assessment of Cancer Therapy – General (FACT-G)**						
Physical Well-being	−.40	−.65*	−.23	−.55	−.43	−.48
Social/Family Well-being	.40	.20	.32	.18	−.22	.08
Emotional Well-being	−.45	−.49	−.08	−.48	−.23	−.59*
Functional Well-being	59*	.76**	.30	.55	.07	.32
Social Concerns	.39	−.71**	−.39	−.61*	−.47	−.48
**Emotional and Spiritual Wellbeing Measures**						
Zung Depression Scale	−.55	−.54	−.37	−.48	−.03	−.28
State-Trait Anxiety Inventory	−.42	−.48	−.37	−.21	−.20	−.17
Spiritual Well-being	.55	.39	.13	.11	−.23	−.07
Spiritual Belief Inventory	.23	.25	−.01	.00	−.12	.00
Bottomley Social Support	−.55	−.37	−.62*	.16	.07	−.14
**Fatigue and Sleep Quality Measures**						
Fatigue Symptom Inventory	−.39	−.83***	−.47	−.54	−.57**	−.50
Pittsburgh Sleep Quality Index	−.40	−.30	−.38	−.72**	−.63**	−.70**
Sleep Hygiene Likert Scale	.24	.79**	.27	.28	.73**	.60*

*p < 0.05; **p < 0.01; ***p < 0.001

**Table 4:** Partial correlation coefficients for neuropsychological measures for active treatment patients

	**Partial Correlation – Controlling for Age & Education**					
	Two Months Into Treatment			After Treatment Completion		
	CD4	CD8	CD16	CD4	CD8	CD16
Code Substitution	−.61*	−.74**	.02	−.20	.00	−.60
Code Substitution Memory - Immediate	−.52	−.57	−.05	.13	−.27	−.17
Code Substitution Memory - Delayed	−.37	−.45	.04	.02	−.21	−.35
Sternberg Memory Recall	.02	−.10	−.33	.04	.06	−.30
Running Memory Continuous Performance	−.58*	−.14	.39	.27	.28	−.12
Logical Reasoning - Symbolic	−.69*	−.46	.16	.31	.28	.08
Spatial Processing - Simultaneous	−.64*	−.42	−.07	.01	.23	−.37
Matching to Sample	−.34	−.14	−.04	−.26	−.31	−.40
Digit Set Comparison	−.60*	−.68*	−.05	.14	.11	−.01
Mathematical Processing	−.62*	−.61*	.05	.09	.39	−.30
Simple Reaction Time	−.36	−.39	−.45	.39	.22	.25

*p < 0.05; **p < 0.01

**Fig. 3:**
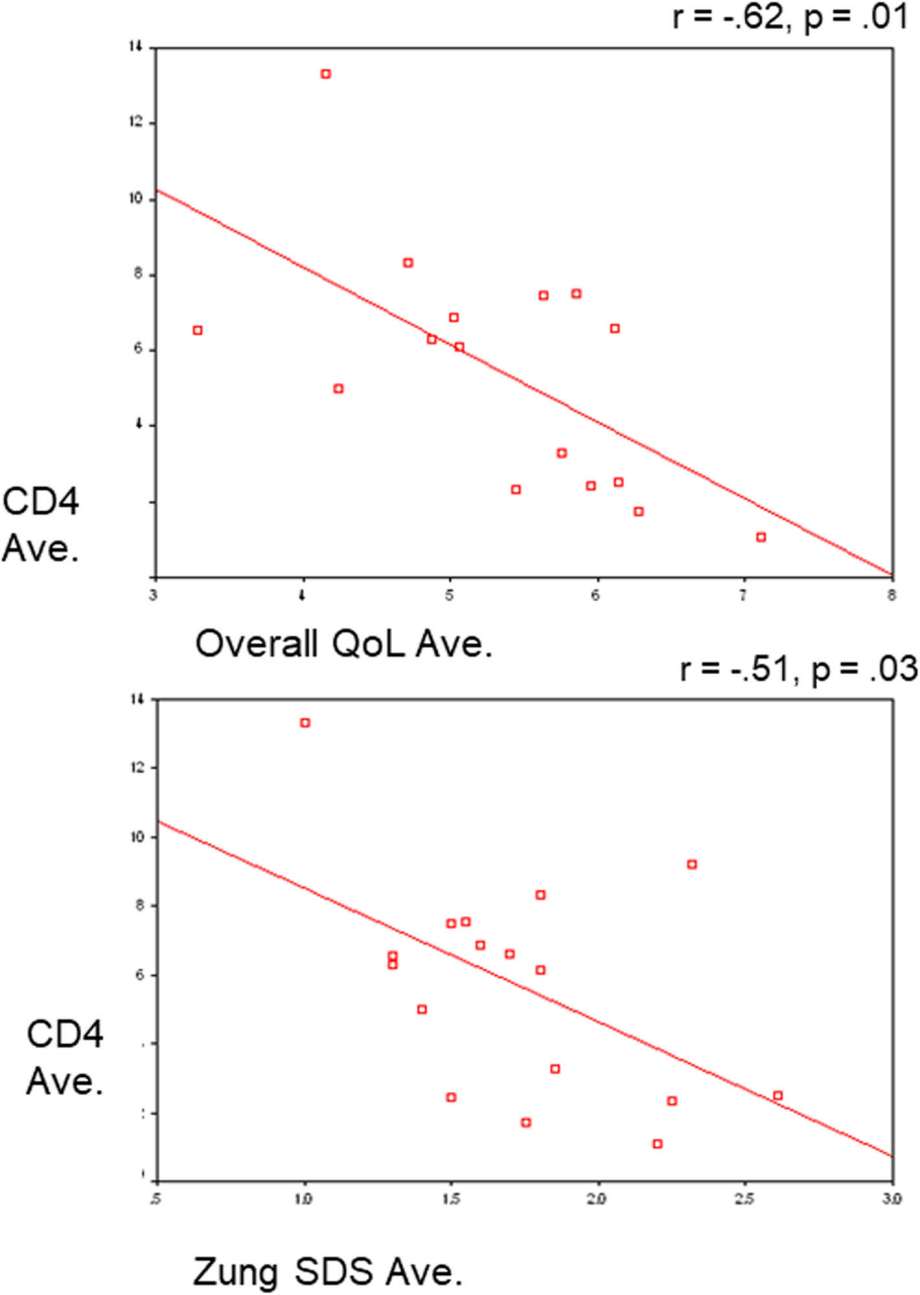
legend. Scatterplots with linear regression least-squares fit for the relationship between CD4 and average mean item scores (adjusted for age at enrollment) for each active treatment patient on the overall Hope Quality of Life (QoL) (upper scatterplot), and between CD4 and mean item average for each active treatment patient on the Zung depression scale (Zung SDS) (lower scatterplot). The Pearson Product Correlation coefficient and corresponding statistical *p* value for the coefficient are noted above each scatterplot

To examine the relationships between the various assessments and the CD4, CD8, and CD16 immunological counts -- a partial correlation was used which controlled for age and education for assessments at months 2 and 4 (during treatment; Table 3). At two-months after diagnosis for the active treatment group, the Hope QoL domains were negatively correlated to CD8 levels except for SWB, in that the higher CD8 levels were during treatment, the poorer the QoL (Table 3). The same was true for the FACT-G domains of physical and functional well-being and concerns about medical status, as well as for the Fatigue Symptom Inventory and Sleep Hygiene Scale (Table 3). For the active treatment group following treatment (4th assessment), CD4 was negatively correlated with CD4 on QoL total (*r* = − 0.62, *p* = 0.010, Fig. 3 upper) and Zung depression (*r* = − 0.51, *p* = 0.026; Fig. 3 lower), in that higher CD4 was associated with poorer QoL and more depression.

For the non-treatment comparison group, there was a statistically significant negative correlation between CD8 and Zung depression at enrollment (r_p_ = − 0.59, *p* = 0.018), and with Fatigue Symptom Inventory (*r* = − 0.51, p = 0.026). In the comparison group, CD16 was positively correlated with QoL Total at enrollment, with higher CD16 corresponding to better QoL (*r* = 0.57, *p* = 0.020). No other statistically significant partial correlation relationships were apparent for the breast cancer survivor (not on active treatment) comparison group at the other assessment points.

For the active treatment group during treatment, higher levels of CD4 and CD8 were related to poorer ANAM neuropsychological performance code substitution, digit set comparison, and mathematical processing speed/accuracy (throughput) (*p* = 0.023; Table 4). CD4 was also negatively correlated with running memory continuous performance, logical reason, and spatial processing (p = 0.02 to *p* = 0.04). CD4 and CD8 were not significantly correlated to ANAM neuropsychological performance at the completion of treatment (4th assessment, Table 4). CD4, CD8, and CD16 were not significantly correlated to ANAM neuropsychological performance for the cancer survivor comparison group of women.

## Discussion

In our study, women undergoing both radiation and chemotherapy showed a significant decline in CD8 levels through the course of treatment compared to radiation treatment only throughout the course of treatment. Furthermore, the higher the level of CD8 among active treatment breast cancer patients during treatment, the poorer the QoL and sleep quality. This was also the case for higher CD4 and CD8 and poorer neuropsychological performance. Figure 2 depicts the decline in CD8 especially for women in the active treatment group undergoing chemotherapy and radiation compared to radiation only following surgery. At the same time, Fatigue Symptoms increases through the course of treatment for the chemotherapy/radiation group compared to radiation only. These comparative trends are sensible, based on our understanding of the psychoneuroimmunology literature. Such relationships are consistent throughout Table 3 in terms of greater symptomology and poorer quality of life being related to lower CD8 response during treatment. On the other hand, less symptomatology, less depression and anxiety and better quality of life, and greater spiritual well-being is related to higher CD4 response (Table 3 and Fig. 2). Most of these relationships diminish following completion of treatment.

Finally, in a University of Pennsylvania nursing doctoral dissertation on “The Effects of Chronic Stress on CD8 T Cells in Human Adults: An Examination from Bench to Bedside”, Christina Marie Slota (2015) concludes that “… Individuals with high levels of norepinephrine (NE) in their serum, or were family caregivers, had a pro-inflammatory state before and after antigenic challenge of memory CD8 T cells. These findings suggest chronically stressed individuals may be more susceptible to previously encountered antigenic challenges compared to novel challenges.” (Abstract, p. 1) [47].

The effects of breast cancer disease and treatment, especially chemotherapy, on neuropsychological functioning have been examined in studies with breast cancer patients over the past couple of decades [48–50]. The cognitive fatigue from the psychosocial stress associated breast cancer disease and treatment may be largely responsible for diminished neuropsychological performance in active treatment patients [10]. However, our study is one of the first to evaluate the relationships among immunological biomarkers, emotional well-being, spiritual well-being, and neuropsychological impact on performance during the course of treatment. Furthermore, we have evaluated these interrelationships among breast cancer survivors at least a year following completion of treatment. Significant interrelationships among these domains exist as well for the remission group. Most of the findings that are discussed in the relevant literature are related to the women undergoing active treatment, which is not new. We now know, however, that there are some women surviving breast cancer that continue to experience cognitive dysfunction even years after finishing treatment [51].

Although our findings are based on a preliminary study with only a small subgroup of active treatment patients undergoing chemotherapy, the use of immunological biomarkers during the course of differing types of breast cancer treatment may help disentangle the effects of the disease itself and its treatment from the psychosocial stress causing cognitive fatigue and diminished capacity [52, 53]. To illustrate, Kenne Sarenmalm et al. [54] analyzed blood samples from patients with breast cancer that had mood disorders. Flow cytometry for NK-cell activity, lymphocyte phenotyping, and ELISA to measure cytokine concentration were used to measure immune response. The results showed positive benefits on the immune system when relieving anxiety with psychotherapeutic interventions [54]. A similar intervention clinical trial could evaluate the extent to which such interventions for alleviating psychosocial stress could enhance neurocognitive function during chemotherapy, as differentiated by immunological biomarkers sensitive to such effects.

For example, Hsaio et al. (2012) observed that body-mind-spirit group therapy sessions maintained stable cortisol levels in response to stressors in breast cancer survivors [55]. Mindful based stress reduction positively influences cytokine production (IL-6 and TNF-alpha) in breast cancer patients [56]. Zhang et al. (2016) was not able to demonstrate statistical significance in the claim that mindfulness based therapy can improve physical and psychological health as an adjuvant therapy in breast cancer patients [57]. But another study showed that mindful based stress reduction significantly improved quality of life and sleep quality. They hypothesized that this was because T-cell and IL-4 production were increased while NK and IL-10 production decreased [58]. This immunological change is common for someone that is emerging from a depressive episode. The use of psychotherapy in combination with medical treatment helps to raise the survival rate of those stricken with breast cancer.

### Spiritual well-being & immune functions

We documented significant relationships between heightened inflammatory immunology biomarkers and poorer City of Hope questionnaire total QoL score, specifically for the active treatment breast cancer patients. However, this relationship did not extend to strong spiritual beliefs or spiritual well-being as measured in our questionnaires. In contrast, Bauer-Wu and Farron (2005) observed in their research that in breast cancer survivors that a positive correlation exists between meaning in life and spirituality [8]. Immune response can improve for persons who gain a greater sense of meaning in life. To illustrate, helper and cytotoxic T-cells were found to be in great numbers when compared to higher levels of spirituality with advanced cancer patients based on spirituality scores using the Rorschach’s test [18].

### Limitations

Since this was a preliminary study on the relationships between the immune system, psychosocial measures, and neuropsychological measures, the sample size was small, so our findings are exploratory in nature with outcomes from many QoL and neurocognitive evaluative domains. Also, we only focused on a few immunological biomarkers from a vast array of possibilities, selecting only those already having some basis in the research for a robust relationship between emotional wellbeing, spiritual wellbeing, and immunological response in patients undergoing treatment for breast cancer and for HIV [11, 16, 59]. Thus, the relationships noted between QoL, neurocognitive performance, and immunological biomarkers are only preliminary at best. At best, our present findings are not conclusive, but establish the viability of evaluating these domains in a more rigorous and better-powered clinical research program with breast cancer patients and survivors.

Due to the small sample size, the relationships observed between CD4 and CD8 levels and poorer City of Hope questionnaire total Quality of Life score, and neuropsychological performance may not extend to all manner of active treatment breast cancer patients experiencing other modes of treatment [60, 61]. None of participants in this study had metastatic disease which could further contribute to our findings on cognitive function if intracranial metastases were reported. Our breast cancer groups were not ethnically diverse in that they were all Caucasian and in their 50s and 60s, living in the Midwest. Therefore, the relationship between immunological biomarkers and emotional or spiritual well-being, for example, may be very different for our women than for women from other ethnicities, cultural contexts, or religions.

Since the study was completed on a voluntary basis at a hospital for cancer treatment and a comparison group of breast-cancer survivors voluntarily participating in a support group, our present findings may also be limited in terms of self-selection bias for those willing to seek psychosocial support, and a sample of cancer patients (past and present) based on availability. This limits the external validity and generalizability of our findings. As such, our present findings based on this preliminary study are only exploratory in nature.

### Future research

The results from this study show that there are some relationships that could be examined further. More research using the same methods with a larger, more representative sample could help to strengthen the results of this study. Once the relationships among these domains are better understood within a more comprehensive analytical model, the causality between the variables can then be explored. The understanding of causality from more comprehensive studies that are prospective in nature will inspire holistic care packages of treatment for the patient with breast cancer, treating the patient as a complete person (mind, body, spirit) as a standard of care [54].

## Conclusion

Our study demonstrates that the treatment of breast cancer is as complex as the disease process itself. It involves not only the treatment of the malignancy in isolation, but rather in concert with psychological and spiritual support and therapy. The results from this study showed that certain immune cells, CD8, could be a useful marker in multiple aspects of quality of life in patients who are battling breast cancer.

## Data Availability

The data that were used in the analyses for the manuscript are not publicly available. They could however be availed upon reasonable request by writing an email to the corresponding author.

## Abbreviations

ANAM: Automated neuropsychological assessment metric
ANCOVA: Analysis of co-variance
ANOVA: Analysis of variance
BCSS: Bottomley cancer social support scale
CBC: Complete blood count
CCCU: Council of Christian Colleges and Universities
CD16: Natural killer (NK) type cytotoxic T cells
CD16/56: Ratio of CD16 NK to CD56 NK receptor-type T cells
CD19: B lymphocyte receptor type 19 cell
CD4: Helper T cells
CD8: Cytotoxic T cells
CMF: Cyclophosphamide methotrexate fluorouracil
ELISA: Immunological assay for labeled immunologically specific antigens
EWB: Emotional well-being
FACS: Fluorescence-activated cell sorting
FACT-G: Functional assessment of cancer therapy - general
fMRI: Functional magnetic resonance imaging
FSI: Fatigue symptom inventory
HIV: Human immunodeficiency virus
HR: Hormone receptor
HER2: Human epidermal growth factor receptor 2
IBM: International business machines
IFN-g: Interferon-gama
IL-1: Interleukin-1 cytokine
IL-10: Interleukin-10 cytokine
IL-12: Interleukin-12 cytokine
IL-2: Interleukin-2 cytokine
IL-4: Interleukin-4 cytokine
IL-6: Interleukin-6 cytokine
IRB: Institution review board for ethics in human research
IUPUI: Indiana University – Purdue University, Indianapolis
MFSI: Multidimensional fatigue symptom inventory
N: Number
NE: Norepinephrine
NK: Natural killer cells
p: Probability
PNI: Psychoneuroimmunology
PSQI: Pittsburgh sleep quality index
QoL: Quality of life
r: Pearson-product moment correlation coefficient
SBI: Spiritual beliefs inventory
SES: Socio-economic status
SPSS: Statistical program for the social sciences (SPSS Inc.)
SRT: Simple reaction time
SSRI: Selective serotonergic re-uptake inhibitor medication
STAI: State-trait anxiety inventory
sTNFr1: Tumor necrotic factor receptor type 1
SWB: Spiritual well-being
TxPA: Toxicity-preventing activity
Zung SDS: Zung self-rating depression scale
